# P30 Bacterial coinfection and prescribing trends in SARS-CoV-2 infection in two UK hospitals

**DOI:** 10.1093/jacamr/dlac004.029

**Published:** 2022-02-16

**Authors:** P. J. Teoh, I. Maan, J. Uwagwu, A. Odedra

**Affiliations:** Queen Elizabeth Hospital, Lewisham and Greenwich NHS Trust, London, UK; Queen Elizabeth Hospital, Lewisham and Greenwich NHS Trust, London, UK; Queen Elizabeth Hospital, Lewisham and Greenwich NHS Trust, London, UK; Queen Elizabeth Hospital, Lewisham and Greenwich NHS Trust, London, UK

## Abstract

**Objectives:**

To describe antibiotic prescribing and early bacterial and fungal coinfection in patients admitted to two London hospitals with COVID-19.

**Methods:**

A retrospective review of adults with PCR-confirmed SARS-CoV-2 infection admitted between 9 February and 10 May 2020. Demographics, critical care unit (CCU) admission, antibiotic prescribing and microbiology results within 10 days of COVID-19 diagnosis were analysed.

**Results:**

In total, 1155 patients were identified; 32.9% (380) died during admission and 12.4% (143) had positive microbiology. After excluding likely contaminants, 6.9% (80) had evidence of coinfection. The most common organisms isolated from blood cultures were *Escherichia coli* 9.5% (7), *Klebsiella pneumoniae* 4.0% (3), and MSSA 2.7% (2). Organisms isolated from lower respiratory tract samples included *Candida albicans* 44.4% (12), *Staphylococcus aureus* 22.2% (6), *Klebsiella* species 11.0% (3), *Pseudomonas aeruginosa* 11.0% (3) and *Citrobacter* species 11% (3). *Legionella* and pneumococcal urinary antigen tests were positive in 0/117 and 2/71 episodes, respectively. Patients admitted to CCU during their inpatient stay were more likely to have positive microbiology compared with patients managed outside CCU (26.2% versus 11.0%, *P<*0.001). Ninety-one percent (1051) of all patients received antibiotics. Clarithromycin (24.2% total antibiotic use) and amoxicillin (21%) were most frequently used, followed by piperacillin/tazobactam (12.6%), gentamicin (10.6%), co-amoxiclav (9.3%) and meropenem (3.2%). Patients given piperacillin/tazobactam or meropenem had a higher length of stay and mortality.

**Conclusions:**

Bacterial coinfection in COVID-19 is uncommon, but more frequent in patients requiring CCU admission. Antibiotic use was widespread, despite lack of microbiological evidence of coinfection. When present, infection was more likely due to Gram-negative bacteria. Future local clinical and antimicrobial guidelines should reflect these findings.

